# Long-term immune reconstitution and T cell repertoire analysis after autologous hematopoietic stem cell transplantation in systemic sclerosis patients

**DOI:** 10.1186/s13045-016-0388-5

**Published:** 2017-01-19

**Authors:** Dominique Farge, Lucas C. M. Arruda, Fanny Brigant, Emmanuel Clave, Corinne Douay, Zora Marjanovic, Christophe Deligny, Guitta Maki, Eliane Gluckman, Antoine Toubert, Helene Moins-Teisserenc

**Affiliations:** 1 Unité de Médecine Interne, Maladies Autoimmunes et Pathologie Vasculaire, UF 04, Assistance Publique Hopitaux de Paris AP-HP, Hôpital Saint-Louis, Paris, France; 2 INSERM UMR-1160, Institut Universitaire d’Hématologie, Paris, France; 3 Université Paris Diderot, Sorbonne Paris Cité, Paris, France; 4 Center for Cell-based Therapy, Regional Blood Center of Ribeirão Preto, Ribeirão Preto, Brazil; 5 Department of Biochemistry and Immunology, Ribeirão Preto Medical School, University of São Paulo, Ribeirão Preto, Brazil; 6 Département d’Hématologie, AP-HP, Hôpital Saint-Antoine, Paris, France; 7 Service de Médecine Interne, Hôpital Pierre Zobda Quitman, Fort-de France, Martinique France; 8 Laboratoire d’Immunologie-Histocompatibilité, AP-HP, Hôpital Saint Louis, Paris, France; 9 Eurocord-Monacord, AP-HP, Hôpital Saint-Louis, Paris, France; 10 Centre Scientifique de Monaco, Monaco, France

**Keywords:** Systemic sclerosis, T cell repertoire, Immune reconstitution, Hematopoietic stem cell transplantation

## Abstract

The determinants of clinical responses after autologous hematopoietic stem cell transplantation (aHSCT) in systemic sclerosis (SSc) are still unraveled. We analyzed long-term immune reconstitution (IR) and T cell receptor (TCR) repertoire diversity in 10 SSc patients, with at least 6 years simultaneous clinical and immunological follow-up after aHSCT. Patients were retrospectively classified as long-term responders (A, *n* = 5) or non-responders (B, *n* = 5), using modified Rodnan’s skin score (mRSS) and forced vital capacity (FVC%). All patients had similar severe SSc before aHSCT. Number of reinjected CD34^+^ cells was higher in group B versus A (*P* = 0.02). Long-term mRSS fall >25% was more pronounced in group A (*P* = 0.004), the only to improve long-term FVC% >10% (*P* = 0.026). There was an overall trend toward increased of T cell reconstitution in group B versus A. B cells had a positive linear regression slope in group A (LRS = 11.1) and negative in group B (LRS = −11.6). TCR repertoire was disturbed before aHSCT and the percentage of polyclonal families significantly increased at long-term (*P* = 0.046), with no difference between groups. Despite improved skin score after aHSCT in all SSc patients, pretransplant B cell clonal expansion and faster post-transplant T cell IR in long-term non-responder/relapsing patients call for new therapeutic protocols guided by IR analysis to improve their outcome.

## Introduction

Systemic sclerosis (SSc) is characterized by progressive fibrosis in the skin and internal organs [1], with 5-year mortality rates up to 30% in rapidly progressive diffuse cutaneous SSc (dcSSc) according to the extent of lung, heart, and kidney involvement [2]. In severe SSc patients, early European and North American phase I–II clinical studies showed that autologous hematopoietic stem cell transplantation (aHSCT) allowed rapid and durable regression of skin and lung fibrosis [3, 4] with improved functional status [5]. Results from the Autologous Stem Cell Transplantation International Scleroderma (ASTIS) multicenter randomized phase III trial demonstrated that aHSCT confers better long-term survival than 12 monthly intravenous pulses of cyclophosphamide in 156 early severe dcSSc patients [6]. With around 1000 SSc patients worldwide transplanted, aHSCT has become the best treatment option for severe rapidly progressive SSc [7].

The rationale for treating autoimmune diseases (AD) with aHSCT involves non-specific abrogation of autoreactivity, mainly T and B cells, followed by reconstitution of a more tolerant immune system and self-protective profile [8, 9], in the ideal context that environmental triggering factors will no longer be effective [10]. Studies in a few AD patients showed that aHSCT acts differently from standard immunosuppression, which modulates specific components of the autoimmunity process, while aHSCT allows to reset the immune response and induces de novo tolerance [7]. Therefore, immunological monitoring is a key element of clinical follow-up post-transplant [11].

We had previously reported the early immune reconstitution (IR) profile associated with clinical remission shortly and up to 1 year after high-dose cyclophosphamide and CD34+-selected aHSCT in seven SSc patients [8]. Recovery of CD3+ T cell reconstitution was delayed with persistent CD4+ T cell lymphopenia. NK cells returned to normal within a month after transplant, while circulating B cell levels were inversely associated with clinical response, suggesting that pathogenic B cell clones might preferentially expand if unfavorable outcome [8]. Thereafter, Tsukamoto et al. found persistent inversion of the Th2/Th1 ratio in eleven SSc patients until 3 years post-aHSCT [12]. In a pilot study, we reported restoration of Tregs and their suppressive function 2 years after aHSCT in seven SSc patients compared to controls [13], and partial fall of the pre-transplant increase in pro-fibrotic and Th2-cytokines serum levels [14]. Several questions remain, such as which of the post-transplant IR mechanisms are most relevant to warrant prolonged remission and what is the duration of the immunological transplant-induced effects?

We therefore designed the present study to analyze the long-term IR, using combined approaches of immunophenotyping and T cell receptor (TCR) diversity analysis according to the observed clinical response in 10 SSc patients before and up to at least 6 years after aHSCT.

## Methods

### Study design and patients

We selected 10 SSc patients treated with aHSCT at Saint-Louis Hospital (Paris, France) and for whom repeated simultaneous clinical and immunological monitoring had been obtained until long-term, at least 6 years, after HSCT. All patients gave a written informed consent. They had received CD34+-selected aHSCT, without or with rabbit antithymocyte globulin (rATG, Genzyme) as part of the previously published ISAMAIR [3] or ASTIS [6] protocols approved by the ethics committee. As a control group, 18 healthy donors were tested to determine the reference values of TCR diversity.

### Transplant procedure

Transplant procedure was previously described [3, 6]. In brief, mobilization and collection of peripheral blood hematopoietic stem cells (PBSC) using cyclophosphamide at 2 g/m^2^/day on two consecutive days followed 4 days later by rHu G-CSF (Lenograstim®, Aventis and Chugai Pharma France) at 5 μg/kg/day subcutaneously until the last apheresis. PBSC were collected when CD34+ cells were above 20/μL in peripheral venous blood and CD34+ cells were selected using immunomagnetic bead technique (Nexel Isolex®300i Stem Cell Collection System). Conditioning was performed at least 4 weeks later, using cyclophosphamide at 50 mg/kg/day from day 5 to day 2 prior to CD34+-selected HSC reinjection without or with rATG. All patients received rHu G-CSF after the graft until neutrophil recovery.

### Clinical follow-up before and after aHSCT

Clinical follow-up was performed as previously described during the first year after aHSCT [8] and thereafter at yearly intervals plus or minus 6 months. Clinical response after aHSCT was assessed by the same observer using repeated functional and physical examination of organ involvement. Relapse was defined by any of the following criteria [15]: an increase of skin score by 25% from best improvement, as assessed by modified Rodnan skin score (mRSS), or a decline in forced vital capacity (FVC, %) by 10%, renal crisis, start of total parenteral nutrition, or restarting of immune suppressive or modulating medication. According to the clinical response at long-term, SSc patients were retrospectively classified as long-term responders (group A) and non-responders or relapse or necessitating immunosuppression (group B) within the first 6 years post-aHSCT. We analyzed their IR profiles and evolution of TCR repertoire according to the clinical outcome after aHSCT.

### Immunomonitoring

At time of each clinical evaluation before and after AHSCT, blood and serum samples were drawn for IR analysis. Prospective lymphocyte immunophenotyping was performed on freshly collected whole blood samples, using a FACS Canto II flow cytometer and FACS DIVA software (BD Biosciences, le Pont de Claix, France). Absolute counts were determined using the TruCount system (BD Biosciences) with BD Multitest (BD Biosciences). Eight color labeling was performed with the following mAbs: CD3, CD4, CD8, CD16, CD56, CD45RA, CD45RO, and CD19. Data were analyzed using FACS Diva (BD Biosciences). Anti-Scl-70 antibody level detection was performed by enzyme-linked immunosorbent assay as previously described [8] and after the year 2010 by BioPlex ANA Screen (Bio-Rad, Hercules, CA).

### T cell repertoire and clonality

Determination of TCR-Vβ usage was made from total RNA using quantitative “Immunoscope” [8]. Briefly, RNA was purified using TriReagent (Molecular Research Center, Cincinnati, OH). Synthesis of complementary DNA (cDNA), complementarity determining region 3 (CDR3) gene amplifications, run-off using an internal β-chain gene constant fluorescent primer, gel running, and Immunoscope software analysis were performed [8]. The definition of Immunoscope profiles as polyclonal, skewed, or negative was based on the identification of peaks that deviate from the normal distribution curve [8].

### Statistical analysis

All results are shown as mean ± standard deviation (SD). Significant differences (*P* ≤ 0.05) between patient groups related to individual conditions were assessed with Mann-Whitney test and ANCOVA for general linear regression analysis, using SPSS software. To reduce intra-group variability induced by individual differences at entry, we used both mean absolutes values and percentages of the total number of cells at inclusion that remained at each time follow-up post-aHSCT for IR analysis, and global trends were expressed using linear regression slope (LRS) [8].

## Results

### Patients clinical characteristics and response to aHSCT

**Fig. 1:**
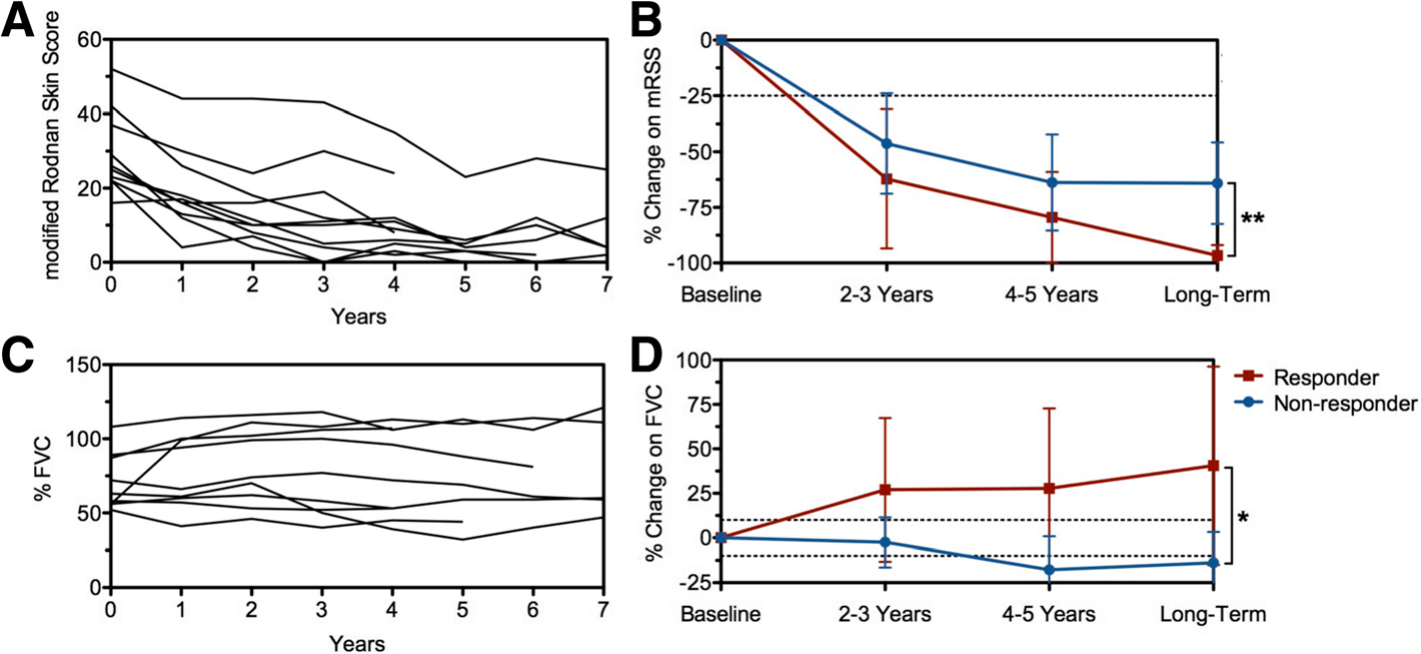
Evolution of modified Rodnan skin score and pulmonary function before and until long-term follow-up (at least 6 years) after aHSCT in SSc patients clinical groups. **a** Evolution of modified Rodnan skin score (mRSS) values in all patients (*n* = 10) from pre-transplant period (0 years) until long-term follow-up (at least 6 years) after aHSCT. **b** mRSS percentage of change (mean ± SD) in the long-term responders (group A, *n* = 5) and non-responders/relapsing (group B, *n* = 5) patients from pre-transplant period (baseline) until long-term follow-up (at least 6 years) after aHSCT. *Dashed line* represents 25% improvement on mRSS. **c** Evolution of Forced Vital Capacity (FVC%) in all SSc patients (*n* = 10) from pre-transplant period (0 years) until long-term follow-up (at least 6 years) after aHSCT. **d** Relative change in Forced Vital Capacity (FVC%) (mean ± SD) in the long-term responders (group A, *n* = 5) and non-responders/relapsing (group B, *n* = 5) patients from pre-transplant period (baseline) until long-term follow-up (at least 6 years) after aHSCT. *Dashed lines* represent improvement (+10%) or worsening (−10%) on FVC. *aHSCT* autologous hematopoietic stem cell transplantation. *P < 0.05 at long-term time-point. **P < 0.01 at long-term timepoint

Ten severe SSc patients, five males, mean age of 37.4 ± 13.9 years, were included in the study. According to the observed clinical response during an overall mean follow-up of 7.8 ± 0.8 years, five patients were long-term responders (group A) and the five others (group B) had either no response or clinical relapse or necessitated immunosuppression post-aHSCT. All were severe dcSSc patients with similar functional status and organ involvement before aHSCT. There was no clinical difference between groups A (*n* = 5) and B (*n* = 5) before transplant, including for mRSS (28 ± 5.7 vs 31 ± 15.2, ns) and FVC% (80 ± 22.2 vs 61 ± 8.8, ns) (Table 1). During the month prior to transplant (baseline), patients received no treatment (*n* = 6) or low dose oral steroids below or equal to 10 mg/day (*n* = 4). The individual patients’ graft characteristics and engraftment duration after CD34+ aHSCT without (*n* = 4) or with rATG (*n* = 6) are in Table 1. The number of reinjected CD34+ cells was higher in group B versus A (4.1 ± 1.3 vs 6.9 ± 2.0, *P* = 0.02), while time to hematopoietic platelets and neutrophils reconstitution were similar in both groups. After transplant, patients received either no treatment up to long-term follow-up (*n* = 5, group A) or mycophenolate mofetil 1–2 g/day (*n* = 5, group B). Low-dose oral steroids below or equal to 10 mg/day were given in three out of five group A and B patients. Compared to pretransplant mRSS values (29 ± 11.0, *n* = 10), all SSc patients had a significant decrease in skin score at 1 year (19 ± 11.1, *P* = 0.029) which was sustained until 6 years (8 ± 9.3, *P* = 0.006, Fig. 1a). The significant regression of skin score, with a fall in mRSS greater than 25% compared to pretransplant values, was present in both groups throughout all follow-up (Fig. 1b) and was significantly more pronounced at long-term in group A than in group B patients (*P* = 0.004, Fig. 1b). Compared to pretransplant FVC values (in % of normal, Fig. 1c), there was no overall change in the lung function post-aHSCT when considering all 10 SSc patients. However, the relative FVC% changes compare to pre-transplant values differed significantly between the two groups during follow-up (*P* = 0.040, ANCOVA), and at long-term, significant improvement of FVC above 10% compared to pretransplant values was observed in group A (*P* = 0.026, Fig. 1d).

**Table 1:** Patients’ clinical characteristics (*n* = 10) with diffuse cutaneous systemic sclerosis (SSc) before autologous hematopoietic stem cell transplantation (aHSCT) and graft characteristics at study inclusion^a^

	Group A patients (*n* = 5)						Group B patients (*n* = 5)						
	1	2	3	4	5	Mean (±SD)	1	2	3	4	5	Mean (±SD)	Mean (±SD)
Patients characteristics at inclusion													
Age, years	40	56	53	47	24	44 ± 12.7	24	27	52	19	32	30.8 ± 12.7	37.4 ± 13.9
Sex	M	M	M	F	F	–	M	M	F	F	F	–	–
Disease duration, months^b^	6	18	29	20	18	18.2 ± 8.2	3	6	27	24	36	19.2 ± 14.1	18.7 ± 10.9
Steroids at inclusion, mg/day	7	10	0	10	0	5.4 ± 5.1	0	7	0	0	0	1.7 ± 3.5	3.8 ± 4.6
SHAQ (0–3)	–	0.87	2.87	1.625	2	1.8 ± 0.8	1.25	2	–	2.125	0.16	1.4 ± 0.9	1.6 ± 0.8
mRSS (0–51)	26	22	37	29	25	27.8 ± 5.7	16	22	52	42	23	31 ± 15.2	29.4 ± 11.0
FVC, %	56	89	87	108	58	79.6 ± 22.2	52	72	63	56	–	60.7 ± 8.8	71.2 ± 19.3
DL_CO,_ % predicted	45	75	72	56	48	59.2 ± 13.7	56	47	72	46	30	50.2 ± 15.4	54.7 ± 14.5
LVEF, %	58	82	62	61	73	67.2 ± 10.0	60	63	–	67	70	65 ± 4.4	66.2 ± 7.7
Serum creatinine, μmol/L	67	76	77	–	51	67.8 ± 12.0	52	73	60	59	71	63 ± 8.8	65.1 ± 10.0
CRP level, mg/L	62	2	15	20	2	20.2 ± 24.7	9	51	66	13	10	35 ± 27.8	26.8 ± 25.6
Anti-Scl-70 antibodies, U/ml	120	+	0	10.2	+	43.3 ± 66.5	0	+	39.1	130	21.3	47.6 ± 57.2	45.8 ± 55.8
Graft characteristics and engraftment duration													
CD34+ cells × 10^6^ infused/kg^c^	4.9	2.9	4.9	2.5	5.3	4.1 ± 1.3	5.8	6.3	5.19	8.79	7.2	6.9 ± 2.0	5.5 ± 2.2
CFU-GM cells × 10^4^ infused/kg^d^	12.37	2.7	–	–	88	34.3 ± 46.7	23	48	9.97	52.56	91	44.9 ± 31.2	40.9 ± 34.7
Days to 0.5 × 10^9^ neutrophils/L	10	10	11	10	10	10.2 ± 0.4	10	7	10	9	9	9 ± 1.2	9.6 ± 1.1
Days to 20 × 10^9^ platelets/L	8	10	10	9	7	8.8 ± 1.3	8	7	10	12	6	8.6 ± 2.4	8.7 ± 1.8
Days to 50 × 10^9^ platelets/L	10	10	13	9	10	10.4 ± 1.5	9	7	Not reached	14	9	9.7 ± 3.0	10.1 ± 2.1

Anti Scl-70 (antitopoisomerase I) antibodies were measured by ELISA and quantified results expressed in arbitrary units (U/ml)

*M* male, *F* female, *SHAQ* Scleroderma Health Assessment Questionnaire, *STE* steroids, *mRSS* modified Rodnan skin thickness score, *FVC* forced vital capacity, *DL*_*CO*_ diffusing capacity for carbon monoxide, *LVEF* left ventricular ejection fraction, *MUGA* multiple gated acquisition scan, *CRP* C-reactive protein. + positive for anti-Scl-70 antibodies

^a^See the “Results” section for description of groups

^b^Calculated since first diagnosis of systemic sclerosis

^c^Quantity of CD34+ progenitor cells contained in the graft. CD34+ cell recovery after cryopreservation was 95% (range 75–100%)

^d^GM-CFUs/kg were counted on day 14 using a clonogenic progenitor assay as previously described[8]

### Phenotypic analysis of lymphocyte populations after HSCT

**Fig. 2:**
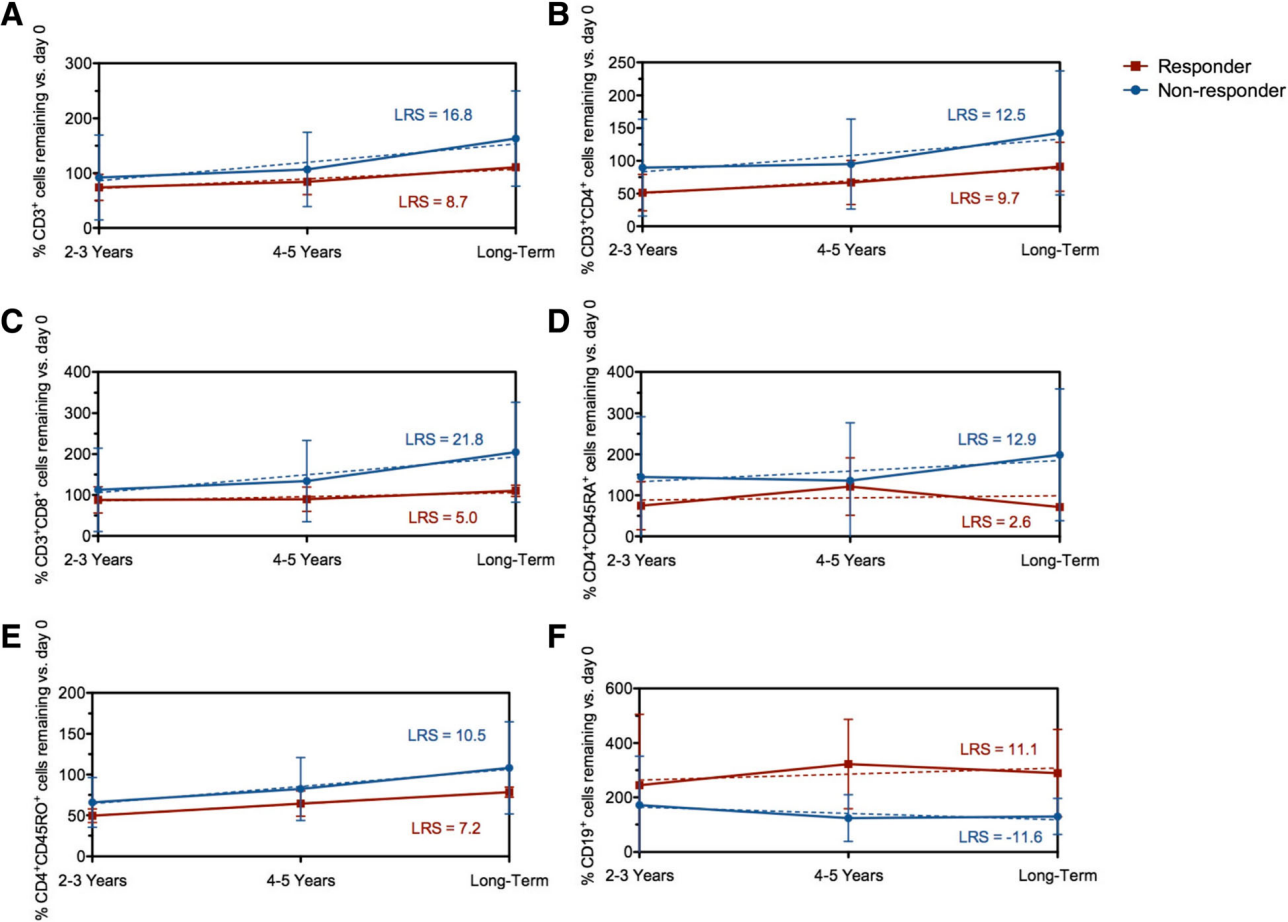
Evolution of lymphocytes immune reconstitution during long-term follow-up (at least 6 years) after aHSCT in SSc patients clinical groups. Results (mean ± SD) are expressed as percentages of the total numbers of lymphocytes at inclusion and the global trends of the immune reconstitution as linear regression slope (LRS) in the long-term responders (group A, *n* = 5) and non-responders/relapsing (group B, *n* = 5) from pre-transplant period (baseline) until long-term follow-up (at least 6 years) after aHSCT. **a** CD3+ total T cells. **b** CD3+CD4+ T cells. **c** CD3+CD8+ T cells. **d** CD4+CD45RA+ naïve T cells. **e** CD4+CD45RO+ memory T cells. **f** CD19+ B cells

At inclusion, the absolute values of T cell subsets, CD4+, CD4+CD45RA+, CD8+ T cells, and number of NK-cells were in normal ranges for all patients and remained stable after aHSCT with no difference between groups A and B (Table 2). The absolute number of B cells was lower in group A than in healthy donors (*P* < 0.05) and was comparable to controls in group B (Table 2). Due to lymphocyte counts variations between patients, we expressed data as percentage of cells compared to numbers before transplant. After aHSCT, there was an overall trend toward increased CD3+ (LRS = 16.8) and CD3+CD4+ (LRS = 12.5) T cell counts in group B compared to group A (LRS = 8.7 and 9.7, respectively) (Fig. 2a, b). A rapid increase in CD3+CD8+ T cell counts starting 2–3 years post-transplant was observed in group B (LRS = 21.8) patients although under immunosuppressive drugs, while they remained almost stable in group A (LRS = 5.0, Fig. 2c). The relative increase in the number of CD4+CD45RA+ was higher in group B (LRS = 12.9) than in group A (LRS = 2.6) patients (Fig. 2d). Memory CD4+CD45RO+ T cell IR profiles were similar in groups A (LRS = 7.2) and B (LRS = 10.5, Fig. 2e). The percentage of B cell change differed with a sustained positive slope in group A (LRS = 11.1), while the slope remained negative in group B (LRS = −11.6, Fig. 2f). Four out of five group A and three out of four group B patients seropositive for anti-Scl-70 before aHSCT became negative after (Table 3). There was no correlation between B cell counts and the presence of high levels of Scl-70 autoantibody (data not shown).

**Table 2:** Phenotypic analysis of lymphocyte population in long-term responders (group A, *n* = 5) and non-responders/relapse or necessitating immunosuppression (group B, *n* = 5) systemic sclerosis patients after autologous hematopoietic stem cell transplantation

Lymphocyte population	Normal range (cell counts/ml)	At inclusion		2–3 years		4–5 years		Long term	
		Group A	Group B	Group A	Group B	Group A	Group B	Group A	Group B
Total	1718–2620	1862 ± 764	1318 ± 748	1348 ± 777	1034 ± 584	1408 ± 937	1135 ± 482	1579 ± 616	1316 ± 624
T cells (CD3+)	1008–1647	1135 ± 485	957 ± 629	937 ± 569	713 ± 477	917 ± 561	749 ± 440	1417 ± 187	1143 ± 527
CD4 T cells (CD3+CD4+)	587–1009	730 ± 163	563 ± 373	409 ± 269	405 ± 305	510 ± 315	412 ± 269	678 ± 345	576 ± 367
Naive CD4 T cells (CD4 + CD45RA+)	161–529	195 ± 161	145 ± 44	197 ± 171	152 ± 221	187 ± 193	158 ± 218	372 ± 175	290 ± 271
CD8 T cells (CD3+CD8+)	313–644	459 ± 182	345 ± 228	453 ± 259	282 ± 171	410 ± 245	299 ± 153	571 ± 39	443 ± 185
B cells (CD19+)	121–267	70 ± 53*	119 ± 91*	300 ± 391	137 ± 137	175 ± 56	208 ± 214	188 ± 63	197 ± 217
NK cells (CD3-CD16+CD56+)	82–340	176 ± 108	112 ± 34	151 ± 64*	92 ± 113*	115 ± 55	132 ± 100	150 ± 92	135 ± 101

Lymphocyte immonophenotyping was performed on fresh whole blood EDTA samples by direct eight-color immunofluorescence flow cytometry. Results are expressed as absolute numbers (mean ± SD) of cell counts/ml

*P = 0.014 between the groups (two-tailed Mann-Whitney)

**Table 3:** Anti-scl-70 autoantibodies

	Group A patients					Group B patients				
	1	2	3	4	5	1	2	3	4	5
Anti-Scl-70 antibodies, U/ml										
Baseline	120	+^a^	0	10.2	+^a^	0	+^a^	39.1	130	21.3
2–3 years	32.9	0	0	0	390	0	240	31.1	106.5	0
4–5 years	11.6	0	0	0	257	0	352	15.9	+^a^	0
Long term	0	0^b^	0^b^	0	>8^b^	0	3.8^b^	>8^b^	250	0

Anti-Scl-70 antibodies were measured at pre-transplant period (baseline) and sequentially during follow-up by enzyme-linked immunosorbent assay as described in methods section. Quantified results are expressed in arbitrary units/ml as previously published ([8])

^a^Positive for Anti-Scl-70 antibodies

^b^Anti-Scl-70 antibodies levels measured by BioPlex ANA Screen

### TCR repertoire diversity

**Fig. 3:**
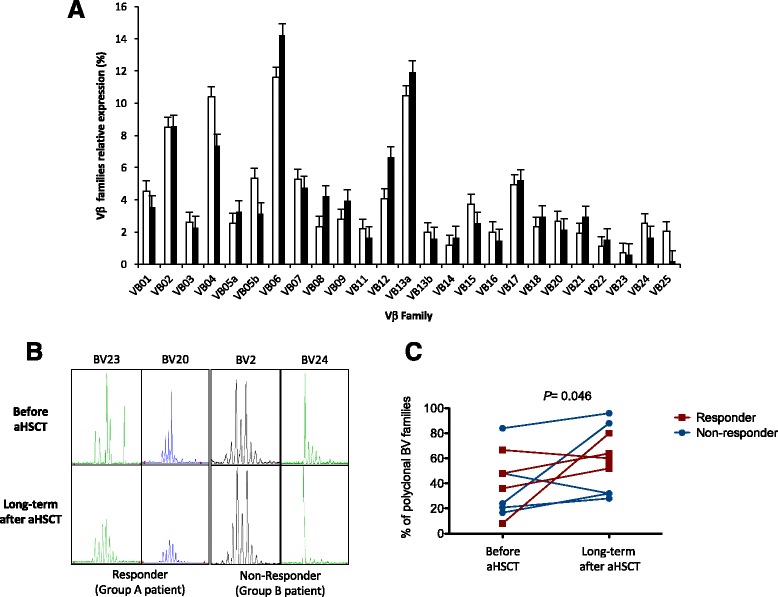
TCR-Vβ family expression and T cell receptor β-chain spectratyping before and at long-term after aHSCT. **a** Quantification of each TCR-Vβ family at baseline (*white bars*) and at long-term (*dark bars*) after aHSCT in 10 dcSSc patients. Data are presented as mean ± SD. There are no differences between the clinical groups. **b** TCR-Vβ chain third complementarity-determining region size distribution profile of selected families at baseline (pre, *upper level*) and at long-term time point (*lower level*) for representative dcSSc patients who underwent autologous hematopoietic stem cell transplantation. *Left*: Polyclonal distribution achievement at long-term time point post-HSCT from a skewed and disturbed repertoire at baseline (patient 3, group A, responder). *Right*: Sustained disturbed distribution at long-term time point post-HSCT from a previously skewed profile at baseline (patient 1, group B, non-responders or relapse or necessitating immunosuppression). **c** T cell repertoire diversity as measured by the percentage of polyclonal TCR-Vβ families in all 10 dcSSc patients at baseline and at long-term follow-up (at least 6 years) after aHSCT

TCR-Vβ family quantifications showed the same relative usage of each family in all the 10 SSc patients before and at long-term after transplantation as observed in healthy subjects (Fig. 3a). As we [8] and others [16] previously reported in SSc patients, the TCR-Vβ family usage did not differ from age-matched controls. CDR3 size distribution was very disturbed with few polyclonal TCR-Vβ families and overexpression of skewed and/or negative families (data not shown). At long-term, distinct changes in repertoire compared to pretransplant TCR-Vβ profiles were observed (Fig. 3b). Two profiles were noted, with either the recovery of a polyclonal profile—similar to healthy individuals—as opposed to a skewed and disturbed repertoire before transplant or the persistence of disturbed profile with still oligoclonaly expanded TCR-Vβ families (Fig. 3b). Overall, T cell diversity improved in almost all long-term patients compared to baseline, and the percentage of polyclonal TCR-Vβ families increased significantly (*P* = 0.046) with no significant difference between groups A and B (Fig. 3c).

## Discussion

Early clinical follow-up of SSc patients after aHSCT has shown rapid and significant improvement in mRSS [5] and improved or stable FVC and DLco on lung function tests [4, 5]. One year after transplant, clinical benefits were such that the North American ASSIST trial closed earlier, after enrollment of 19 instead of 60 SSc patients initially powered, due to failure to reach equipoise between aHSCT and the control group [4]. In the European Society for Blood and Marrow Transplantation (EBMT) ASTIS trial, despite increased treatment-related mortality during the first year, treatment responses in SSc clinical outcome variables 2 years after aHSCT were higher than controls, allowing superior event-free and overall survival rates until 10 years. While all SSc patients selected for transplant had severe disease at entry, early clinical responses at 1 [5, 8] or 2 [6] years after aHSCT as well as baseline cardiac function [15] were shown to predict long-term clinical response.

In the present study, long-term clinical response was not related to disease severity before aHSCT, contrary to seven SSc patients previously analyzed for aHSCT response at 1 year [8]. This may be related to improved patient selection before transplant, while gaining knowledge in the field over the years and following updated EBMT guidelines [7, 15]. Meaningful differences were detected between the long-term responders and non-responders/relapsing patients according to the trends in early SSc clinical response and to global trends of IR. Of note, long-term improvement in skin score was obtained in all 10 patients with a mRSS fall >25%, which was more pronounced in group A patients, who were the only to improve FVC% above 10% after 6 years follow-up. Interestingly, when the two groups of long-term patients were analyzed, the early clinical trends concerning the relative improvements in mRSS and FVC (%) at 1 year after transplant became significant at long-term after transplant. These data suggest that evolution of clinical scores within the early years after transplant indicate long-term clinical responses.

Depending on the conditioning regimen and the underlying disease, several mechanisms contribute to the IR process after aHSCT, which duration vary according to individual patients [10]. During the early phase of IR, the re-emergence of naïve T and B cells, the renewal of the immune repertoire and reinstatement of synergistic immunoregulatory mechanisms are expected [11], but no study had yet evaluated the long-term IR after aHSCT in SSc patients. We also aimed to clarify if maintenance or rapid reintroduction of immunosuppression after transplant, as previously suggested [3], may improve patient outcome despite no response or relapse.

In these 10 severe SSc patients followed for long term after aHSCT, T, B, and NK cells were found within normal ranges before transplantation [8, 12, 13]. After aHSCT, the reconstitution of CD8+, CD4+CD45RA+, CD4+CD45RO+ T, and NK cells was achieved after 2–3 years, confirming previous trends at 1 year [8]. The relative increase in CD4+CD45RA+ after transplant showed sustained activation of the immune system in non-responders/relapsing patients. The same trends in IR were observed for all T cell subtypes, and of note, the CD3+CD8+ increase was steeper in the non-responders as compared to the responder group. This may reflect the persistence of an underlying disease mechanism in these patients [8] and call for new therapeutic protocols after transplant or use of adjuvant cellular therapy [3], such as mesenchymal stromal cells infusion, in order to damper the autoimmune and inflammatory response [17]. Delayed CD4+ T cell recovery was more pronounced in group A than in group B and was sustained at long-term. Further studies will help to decipher the complex interplay between CD4+ T cell subsets and their influence on post-transplant response in SSc and to better elucidate the role of stem cell memory T cells during IR after aHSCT [18].

Our results also suggest that pathogenic B cell clones preferentially expand before transplant in these SSc patients with less favorable outcome at 1 year and thereafter, as previous reported [8]. There was no correlation between the B cell counts and the anti-scl-70 autoantibody levels after aHSCT. However, four out five from group A patients and only one out five from group B patients became seronegative for anti-scl-70 at long-term, illustrating sustained autoimmunity in non-responders or relapsing patients despite reintroduction for immunosuppressive drugs. Nonetheless, the responder patients presented a sustained and positive B cell reconstitution slope, which underlines the need for further refined analysis of the respective number and function of the different B cells subsets, notably the regulatory B cells.

The TCR-Vβ repertoire at baseline was disturbed in all dcSSc patients compared to controls, with a higher number of families presenting a skewed and oligoclonaly expanded profile as previously reported [8, 16]. Here, we show sustained and higher clonal diversity of the TCR repertoire at long-term after transplant in both groups, irrespective of their long-term clinical response. Some oligoclonally expanded families were still found at long-term after transplant both in group A and B patients, indicating that either the patients residual T cells survived the conditioning or were re-infused with the graft at time of aHSCT and can persist for long term or that thymic rebound was not appropriately achieved. As thymic reactivation participate to the observed clinical response after aHSCT [8–10, 19], adjuvant therapies to support hematopoiesis and thymic output could be helpful to improve long-term clinical response [17, 20, 21].

In conclusion, despite improved skin score early after transplant in all SSc patients, pretransplant B cell clonal expansion and faster T cells IR after aHSCT were specific to long-term non-responder/relapsing patients. Immune reconstitution analysis will guide the clinicians for establishing new therapeutic protocols in long-term non-responding/relapsing patients after HSCT.

## Abbreviations

AD: Autoimmune diseases
aHSCT: Autologous hematopoietic stem cell transplantation
CDR3: Complementarity determining region 3
FVC: Forced Vital Capacity
IR: Immune reconstitution
LRS: Linear regression slope
mRSS: Modified Rodnan skin score
SSc: Systemic sclerosis
TCR: T cell receptor
